# Comprehensive Genomic Profiling and Therapeutic Implications for Patients with Advanced Cancers: The Experience of an Academic Hospital

**DOI:** 10.3390/diagnostics13091619

**Published:** 2023-05-03

**Authors:** Laure-Anne Teuwen, Evelyne Roets, Pieter D’Hoore, Patrick Pauwels, Hans Prenen

**Affiliations:** 1 Department of Oncology, Antwerp University Hospital, Drie Eikenstraat 655, 2650 Edegem, Belgium; laure-anne.teuwen@uza.be (L.-A.T.); evelyne.roets@hotmail.com (E.R.); pieter.fn.dhoore@gmail.com (P.D.); 2 Department of Pathology, Antwerp University Hospital, Drie Eikenstraat 655, 2650 Edegem, Belgium; patrick.pauwels@uza.be; 3 Center for Oncological Research (CORE), Integrated Personalized and Precision Oncology Network (IPPON), University of Antwerp, Universiteitsplein 1, 2610 Wilrijk, Belgium

**Keywords:** advanced cancer, genomic profiling, next-generation sequencing, OncoKB classification, targeted therapy

## Abstract

Next-generation sequencing (NGS) can be used to detect tumor-specific genomic alterations. This retrospective single-center study aims to assess the application of an extensive NGS panel to identify actionable alterations and initiate matched targeted treatment for patients with advanced cancer. We analyzed genomic alterations in solid tumor biopsies from 464 patients with advanced cancer with the Foundation Medicine assay (FoundationOne^®^CDx). Therapeutic implications were determined using the Memorial Sloan Kettering Precision Oncology Knowledge Base (OncoKB) classification. The FoundationOne^®^CDx was successfully applied in 464/521 patients (89%). The most common altered genes were TP53 (61%), KRAS (20%), CDKN2A (20%), TERT (16%), and APC (16%). Among the 419 patients with successfully analyzed tumor mutational burden (TMB), 43 patients presented with a high TMB (≥10 mutations/megabase). Out of the 126 patients with an actionable target, 40 patients received matched treatment (32%) of which 17 were within a clinical trial. This study shows that the application of NGS is feasible in an academic center and increases the detection of actionable alterations and identification of patients eligible for targeted treatment or immunotherapy regardless of tumor histology. Strategies such as early referral for NGS, inclusion in clinical (basket) trials, and the development of new targeted drugs are necessary to improve the matched treatment rate.

## 1. Introduction

Next-generation sequencing (NGS) allows high-throughput sequencing of many nucleotides at a fast speed [1]. Application of large multigene NGS panels on tumor tissue has increased the identification of actionable genomic alterations, thereby leading to additional treatment options for patients and the development of the so-called “precision medicine” [2,3,4,5]. Examples of successful pan-cancer targeted therapies include the neurotrophic tyrosine receptor kinase (NTRK) inhibitors entrectinib and larotrectinib in solid tumors with NTRK gene fusions and the fibroblast growth factor receptor (FGFR) inhibitor erdafitinib in FGFR-altered solid tumors [6,7]. Importantly, it has been shown that genotype-matched treatment options may lead to a survival benefit in several cancer types [8].

While smaller sequencing panels are being applied broadly, they require assumptions about possibly present alterations in each tumor type and thereby do not allow the detection of rarer genomic alterations [9]. This can be overcome by the application of large multigene NGS panels. However, the default execution of a large NGS panel in every oncological patient is not yet standard practice for a number of reasons. First, the low frequency of some actionable genomic alterations results in a low likelihood of identifying an actionable genomic alteration in an individual patient [10,11,12,13]. Second, for many targetable genomic alterations, no approved or investigational agent is available. Third, as extensive NGS panels can reveal several genomic alterations, the distinction between ‘true drivers’ versus ‘bystanders’ becomes more challenging [14]. Other critical factors are the cost of sequencing, the time needed for data interpretation, and the cost of targeted therapy [13]. Consequently, most studies show low rates for genotype-matched therapies after performing NGS, and more studies are needed in order to portray to which extent the broad application of NGS could enhance genotype-matched treatment and improve the outcome of cancer patients [15,16,17,18].

In the past years, several NGS testing platforms have been developed, each assessing different types and numbers of genes [19]. The FoundationOne^®^CDx panel was the first Food and Drug Administration (FDA)-approved broad NGS panel validated for all solid tumors [20,21]. The panel analyzes 324 genes as well as tumor mutational burden (TMB) and microsatellite instability (MSI) [22]. Shortly afterward, the MSK-IMPACT (Memorial Sloan Kettering-Integrated Mutation Profiling of Actionable Cancer Targets) test was FDA-cleared [23]. This test initially analyzed 341 genes but has been expanded to 505 genes [24,25]. Meanwhile, other multigene NGS panels such as CANCERPLEX and OmniSeq Advance have been developed. These panels can be applied to multiple tumor types; however, they are not FDA-approved or FDA-cleared [26,27,28].

Here, we applied the FoundationOne^®^CDx panel to 464 oncologic patients with locally advanced or metastatic solid tumors in order to depict how NGS can contribute to treatment options in an academic hospital setting and explore its clinical applicability. This retrospective study aims to describe the genomic alterations detected, the percentage of actionable targets, and the matched treatment rates.

## 2. Materials and Methods

### 2.1. Patients

We retrospectively collected detailed clinical data from patients that were internally or externally referred to the University Hospital of Antwerp by their attending oncologist for next-generation sequencing. Patients with any type of histologically confirmed solid tumor, of which tumor tissue was sent for FoundationOne^®^CDx panel testing between October 2018 and December 2021, were included. For each patient, data were extracted from the Foundation Medicine^®^ report, as well as medical charts, and were cross-checked. Medical chart information included patient demographics, cancer diagnosis, programmed death-ligand 1 (PD-L1) expression (if reported), and treatment with targeted therapies. Data were stored in an anonymized database.

### 2.2. Tumor Samples

Tumor samples were obtained at initial diagnosis, at primary tumor relapse, or at the time of metastatic spread. For each patient, the most recently available tumor tissue was used. In the case of an absence of available tumor tissue, a fresh biopsy was taken. There was no limit in terms of tissue age. All tumor tissue was formalin-fixed paraffin-embedded (FFPE) prior to analysis. In the case of a failed FoundationOne^®^CDx panel testing, information about the cause of failure was collected and categorized as (i) failure to reach minimum quality metrics control, (ii) insufficient tumor tissue, (iii) failure to reach minimum tumor nuclei, and (iv) insufficient amount of deoxyribonucleic acid (DNA) extracted.

### 2.3. Next Generation Sequencing Panel

FoundationOne^®^CDx is a diagnostic test that is based on qualitative next-generation sequencing and uses targeted high-throughput hybridization-based capture technology on DNA, isolated from FFPE tumor tissue to detect insertion and deletion alterations, substitutions, and copy number alterations in 324 genes, as well as certain gene rearrangements and genomic signatures such as tumor mutational burden and microsatellite instability. It was developed as a companion diagnostic to identify targetable mutations in patients who may benefit from treatment with targeted therapies. The determination of MSI is based on a genome-wide analysis of 95 microsatellite loci. TMB is measured based on the total number of synonymous and non-synonymous variants that are present at ≥5% allele frequency and is expressed as mutations per megabase (mut/Mb).

### 2.4. Endpoints and Statistical Analysis

The endpoints for data analysis were (i) the proportion of patients for whom the Foundation Medicine^®^ report had a direct impact on the treatment received, i.e., administration of a treatment associated with the Foundation Medicine^®^ result, (ii) descriptive analyses of the successfulness of Foundation Medicine^®^ testing, mutations discovered, amount of gene alterations per patient, and occurrence of high TMB. All statistical analyses were descriptive and performed with Microsoft Excel software as well as R Studio version 1.2.1335.

## 3. Results

### 3.1. Demographics

**Figure 1:**
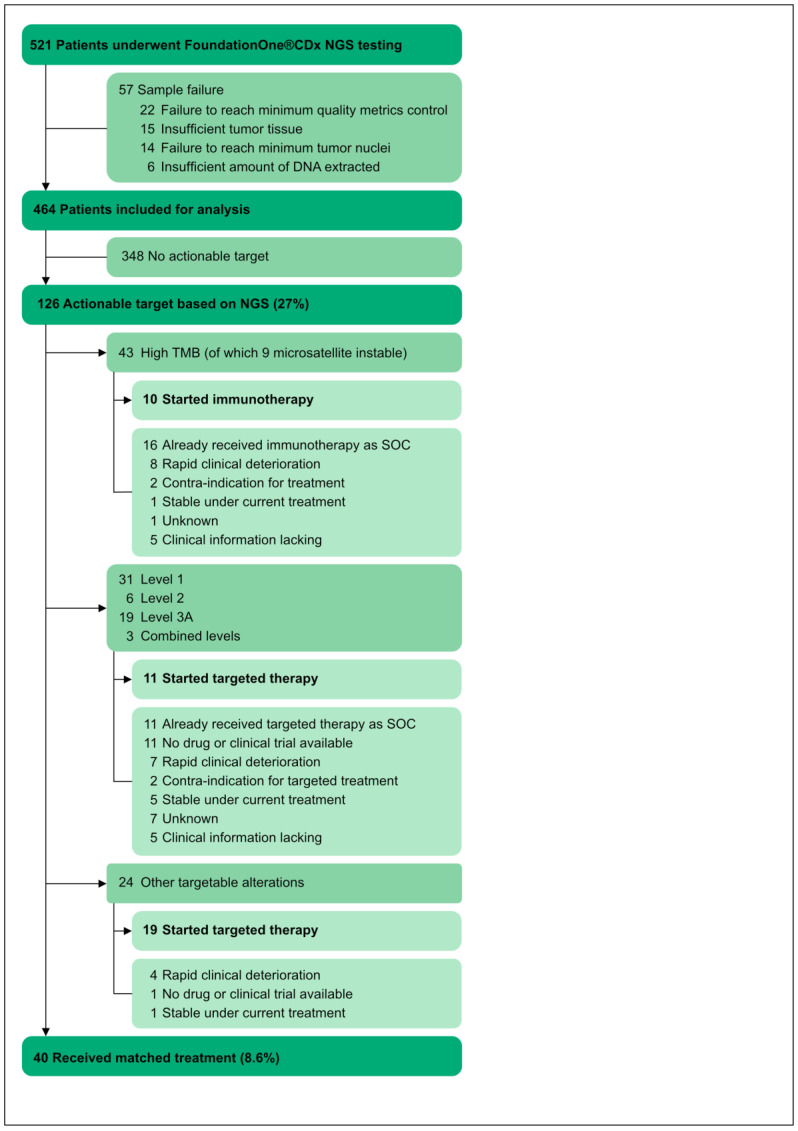
Study flow chart. DNA, deoxyribonucleic acid; NGS, next-generation sequencing; SOC, standard of care; TMB, tumor mutational burden.

In total, 521 patients underwent Foundation Medicine^®^ testing on tissue biopsies between October 2018 and January 2022. Fifty-seven patients were excluded due to sample failure (Figure 1). Reasons for sample failure were failure to reach minimum quality metrics control in 4.2% of the total population, insufficient tumor tissue available in 2.9%, failure to reach minimum tumor nuclei in 2.7%, and an insufficient amount of DNA extracted in 1.2% (Figure 1). Of the 464 tests included for analysis, the median time between tissue biopsy and analysis was 32 weeks (range 0–876 weeks) (Table 1). Overall, 58% of samples were derived from primary tumor tissue and 41% from metastatic tissue (Table 1). At the time of biopsy, 33% of patients were pretreated, while 63% were treatment-naïve (Table 1).

**Figure 2:**
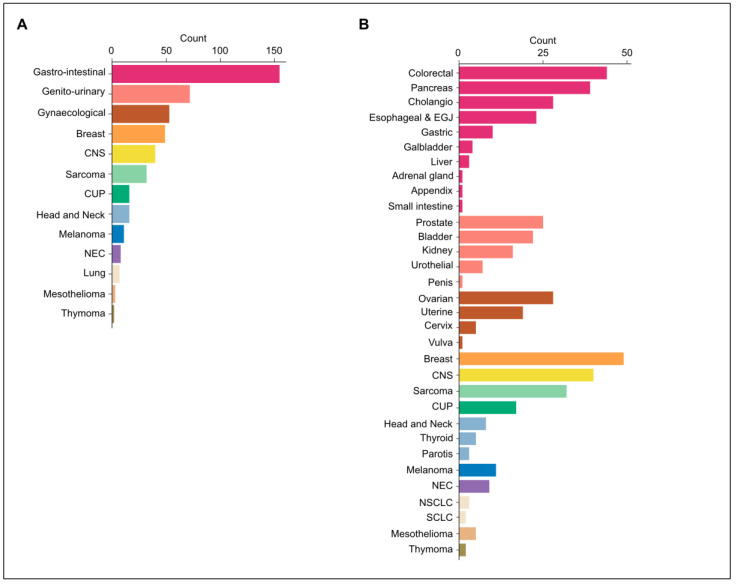
Distribution of tumor types and frequency of gene alterations. (**A**) Distribution of patients included in the analysis according to tumor groups. (**B**) The median number of gene alterations per patient for each tumor group. CNS, central nervous system; CUP, carcinoma of unknown primary; EGJ, esophageal and esophago-gastric junction; NEC, neuro-endocrine carcinoma; NSCLC, non-small-cell lung cancer; SCLC, small-cell lung cancer.

The patient characteristics are summarized in Table 1. Of the 464 patients included in the analysis, the median age at the time of tissue biopsy was 63 years old (range 19–88), and 49% were female patients. Most patients that underwent Foundation Medicine^®^ testing had metastatic disease (72%). The most common tumor group was gastro-intestinal (GI), given the large population of patients with digestive tumors in the referral center (Figure 2A, Table 1). The most common tumor types were breast cancer (11%), colorectal cancer (10%), central nervous system tumors (9%), pancreatic cancer (8%), sarcoma (7%), cholangiocarcinoma (6%), and ovarian cancer (6%) (Figure 2B, Table 1). A summary of all included tumor types is listed in Table 1.

### 3.2. Altered Genes and Mutations

**Figure 3:**
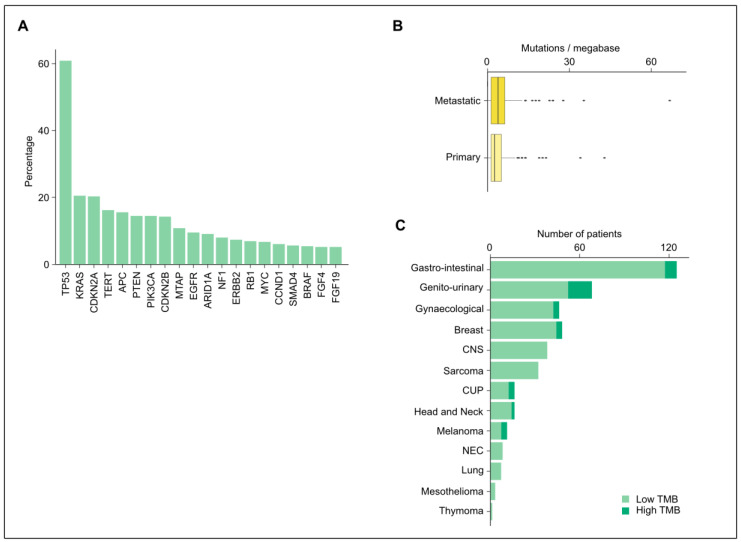
Gene alterations and tumor mutational burden. (**A**) Frequency of genomic alterations across all tumor types (*n* = 464). TP53 was the most frequently altered (61%), followed by KRAS (20%), CDKN2A (20%), TERT (16%), and APC (16%). The percent indicates the percentage of patients with an alteration. The top 20 altered genes are shown. (**B**) TMB according to the tissue of origin. (**C**) The number of patients with high versus low TMB (cut-off 10) according to tumor groups. CNS, central nervous system; CUP, carcinoma of unknown primary; NEC, neuro-endocrine carcinoma; TMB, tumor mutational burden.

In 464 Foundation Medicine^®^ tests, a median of 4 genomic alterations per patient was detected (range 0–19) (Supplemental Table S1). Across all tumor types, TP53 was the most commonly altered gene (61%), followed by KRAS (20%), CDKN2A (20%), TERT (16%), APC (16%), PTEN (14%), PIK3CA (14%), CDKN2B (14%), MTAP (10%), and EGFR (10%) (Figure 3A).

### 3.3. Tumor Mutational Burden and Microsatellite Instability

In the 419 patients (90%) with successfully analyzed tumor mutational burden (TMB), the median TMB was 2.52 (range 0–67) mutations/Mb (mut/Mb) (Supplemental Table S1). In metastatic tissue, the median TMB was 3.78 mut/Mb (range 0–66.8) compared with 2.52 mut/Mb (range 0–42.9) in primary tumor tissue (Figure 3B).

High TMB has been defined as ≥10 mut/Mb, and tumors with a high TMB generally show higher response rates to immunotherapy [20,21,22,23]. In this cohort, a total of 43 patients (10%) presented with a high TMB (Figure 3C). Out of these patients, 10 patients (23%) started immunotherapy based on the TMB result, of which 9 were within a study context. Sixteen patients (37%) were already receiving immunotherapy as standard of care treatment, eight (19%) were not able to consider or start immunotherapy because of rapid clinical deterioration, two (6%) had a contra-indication for immunotherapy, one (2%) was stable under current treatment, and one patient (2%) did not start immunotherapy. For the remaining five patients (12%), clinical information was lacking.

Nine patients (2%) were classified as microsatellite instable (MSI-H). Out of these nine patients, four received immunotherapy within a study context, two were already receiving immunotherapy as standard of care, and two had rapid progressive disease. All patients with microsatellite instability in this cohort displayed a high TMB.

### 3.4. Targeted Therapy Based on NGS Results

The Precision Oncology Knowledge Base (OncoKB) is a database that classifies gene alterations and tumor-type specific treatment implications at different levels of evidence [29]. Level 1 signifies that an FDA-approved indication exists, level 2 signifies standard care, level 3A signifies clinical evidence, and level 4 signifies biological evidence. In our study cohort, 31 patients (6.7%) had a level 1-identified gene alteration, 5 patients (1.3%) had a level 2-identified gene alteration, 19 patients (4%) had a level 3A-identified gene alteration, 100 patients (21.6%) had a level 4-identified gene alteration, and 5 patients had more than one identified gene alteration (level 1 and 3A, level 1 and 4, level 3A and 4, and twice level 4 and 4). A list of the gene alteration and their OncoKB score per tumor type is listed in Table 2. Out of the 59 patients with a level 1–3A gene alteration, 11 patients started a targeted treatment based on the NGS result, of which 5 were within a study protocol, i.e., tucatinib for an ERBB2-amplified colorectal cancer, sotorasib for a KRAS G12C-mutated rectal carcinoma, inavolisib for PIK3CA-mutated breast cancer, ipatasertib for AKT1-mutated breast cancer, and erdafitinib for an FGFR2-rearranged cholangiocarcinoma. Eleven patients were already receiving targeted therapy as a standard of care treatment. For another eleven patients, targeted treatment was unavailable (e.g., for IDH1 R132-mutated tumors), and seven patients deteriorated rapidly after the NGS panel, before targeted therapy could be started (Figure 1).

In addition, 24 patients (5.2%) who were not identified with OncoKB within level 1-3A, were eligible for targeted therapy based on their NGS result, and 19 started treatment. Most patients (*n* = 17) received treatment within a study protocol. Eight patients with a diagnosis of colorectal, pancreatic, esophageal, breast, thyroid, gastric, or cholangiocarcinoma had an FGFR2 alteration and received erdafitinib or derazantinib. Four patients diagnosed with ovarian cancer, melanoma, sarcoma, or cholangiocarcinoma received afatinib based on an EGFR or ERBB3 mutation. Two patients had a BRAF alteration for which they received encorafenib and binimetinib, and cobimetinib, respectively. One patient with an HRAS-mutated thyroid neuro-endocrine tumor received tipifarnib, two patients with a BRCA1-mutated ovarian or cholangiocarcinoma received niraparib or olaparib, one patient with a gastric adenocarcinoma received abemaciclib because of a CDK4 amplification, and one patient with a KRAS G12C-mutated carcinoma of unknown primary received sotorasib.

In total, 35 patients (7.5%) received a new matched treatment based on the NGS result (Figure 1), while 27 patients (5.8%) were receiving, or already received, matched treatment as standard of care.

## 4. Discussion

Broad NGS panels such as the FoundationOne^®^CDx panel allow the detection of a large variety of potentially actionable targets [19]. In this study, we retrospectively analyzed NGS panels from 521 patients with locally advanced or metastatic solid tumors and classified the clinically actionable alterations according to the OncoKB classification [29]. The most commonly altered genes were TP53, KRAS, CDKN2A, TERT, and APC, which is in line with previous reports [8,15,30,31].

The success rate of the FoundationOne^®^CDx panel in this study was 89.1%, despite tumor biopsies with a maximum age of up to 876 weeks. This success rate is similar to the results of previous studies [15,31,32]. Out of the 464 patients with a successful molecular analysis, for 126 patients (27%), an actionable target was identified, which led to a direct change in therapy for 40 patients (8.6%). This percentage is in line with results from other trials. In the study by Karol et al., in 26.4% of patients, a mutation with level 1 and/or 2 and/or 3A, according to OncoKB, was identified, and for 8 patients (12%), this implied a direct change in therapy [15]. In the PROFILER trial, 27% of patients displayed a targetable genomic alteration and 6% received matched treatment [33]. However, several other studies described higher rates for actionable genomic alterations [16,34,35]. These differences could at least partially be explained by the definition of an actionable genomic alteration, which is not uniform across studies [29,36].

Although NGS allows the detection of many genomic alterations, genotype-matched therapy rates remain low, which can be explained by several factors. First, many actionable genomic alterations occur in very low frequencies, which implies a low likelihood to identify an actionable genomic alteration in an individual patient [37]. Second, targeted drugs are often non-existing or inaccessible, because of a lack of reimbursement for the indication, and are only available via a clinical trial or in a compassionate use program [17,29,38]. Third, the level of evidence for a targetable genomic alteration relies not only on the actionable alteration itself but also varies according to the tumor type [29]. Fourth, patients may have further progressed or died during the waiting time for the panel results, especially when the panel is requested after progression on all standard therapies. In this study, the rate of targeted therapy might have been underestimated for the following reasons: (i) Eleven patients received targeted therapy as standard of care before the FoundationOne^®^CDx panel was performed (e.g., anti-HER2 treatment in the case of ERBB2 amplification in nine patients). In those cases, the panel did not identify a new targetable mutation as such. (ii) In eleven patients, a targetable alteration was detected, but no drug or clinical trial was available. Finally, (iii) some patients had rapidly progressive diseases and were therefore not able to receive targeted treatment. This stresses the importance of performing NGS at an early stage of the disease.

One way to improve the matched treatment rate is by setting up basket trials that assess the effect of a certain drug in tumors harboring the same genomic alteration [39]. Increased enrollment of patients in basket trials not only leads to more insight into the genomic spectrum of multiple cancer types but also decreases the costs associated with performing NGS and the resulting matched treatment [40]. A number of trials are ongoing. In the NCI-MATCH, NCT02693535 (TAPUR), NCT04341181 (ProTarget), NCT04185831 (MEGALiT), and NCT05058937 (BALLETT) trials, patients are allocated to a subprotocol based on their tumor genetic profile [32,41,42,43,44,45]. The NCT02152254 (IMPACT 2) trial aims to compare the progression-free survival of patients randomized into a matched targeted treatment arm versus a standard of care treatment arm, and the NCT05385081 (PRECODE) trial is investigating the turnaround time of genomic profiling and frequency of biomarker-driven matched targeted treatment with a prospective manner [46,47]. The PROFILER 02 trial aims to compare the benefit of a large NGS panel with a more limited one [48]. In the CAPTUR trial, patients are enrolled into a treatment arm based on the tumor type and the type of actionable genomic alteration [49].

Since a substantial number of patients were treated within a clinical trial protocol, survival outcomes could not be disclosed. Therefore, this study did not aim to describe the clinical benefit of the broad application of NGS. In other trials, conflicting results have been reported regarding clinical benefits. In the MOSCATO 01 trial, a clinical benefit, defined as progression-free survival (PFS) of 1.3-fold longer than the PFS with prior therapy, was seen in 33% of patients receiving matched treatment (6.6% of the total population) [50]. In the MAST study and in the NCT03089554 trial, a significantly longer PFS was seen in patients receiving matched treatment compared to those receiving standard treatment [31,51]. In the DRUP trial, a clinical benefit rate (complete response, partial response, or stable disease) of 33% was shown [52]. In contrast, in the randomized controlled trial SHIVA, no improvement in PFS was seen in patients receiving matched treatment [35].

This study included a heterogeneous and diverse group of patients with locally advanced or metastatic solid tumors, based on internal or external referral from the attending oncologist. In the literature, no consensus has yet been reached on when to consider an NGS test in a particular patient [12]. Some authors prefer a conservative approach with NGS testing only to be applied within research environments or in the context of a clinical trial [53,54,55]. Other authors advocate for the application of NGS testing on all patients with metastatic tumors who have limited standard of care options, as this approach may identify more potential targets and translate into a higher likelihood of obtaining additional treatment options with an effective matching drug [56,57]. Additionally, NGS testing can be appropriate for navigating patients with specific genomic alterations to clinical trials. Before ordering an NGS test, it remains important for clinicians to ensure that the result of the test will impact the therapeutic plan [12]. MSI and TMB statuses are approved biomarkers to select patients that can benefit from immune checkpoint inhibitors [58,59]. In this study, based on a high TMB and/or MSI, a subset of patients started immunotherapy within or outside of a clinical trial. In the KEYNOTE-158 study, a phase 2 trial that included patients with different tumor types, patients with a high TMB (≥10 mut/MB) displayed higher response rates after treatment with pembrolizumab than patients with a low TMB [58]. These results support the use of MSI status and TMB status regardless of tumor histology. Further research into tumor-agnostic biomarkers to identify patients eligible for immunotherapy or targeted therapy will be important to guide the prescription of these agents.

This study has several limitations. First, the performance of NGS depended on the referral of the attending oncologist. Second, some tumor types were underrepresented (e.g., lung cancer) since small-panel molecular testing is part of standard of care in Belgium for certain tumor types. Therefore, this study did not allow us to compare the percentage and type of actionable genomic alterations between tumor subgroups. Third, this study represents data from patients that were treated in an academic hospital or referred to an academic hospital with access to a range of innovative clinical trials. Fourth, as some patients received targeted therapy within a clinical trial protocol, we could not disclose and discuss survival outcomes.

In conclusion, in this study, we showed that the application of the FoundationOne^®^CDx panel upon physician’s indication led to the identification of an actionable genomic alteration in 27% of patients. Out of these 126 patients, 40 (32%) received matched treatment, which shows that large-panel NGS thereby enabled additional treatment options for an important subset of patients. Almost half of these patients (42.5%) received treatment within a study protocol, which emphasizes the importance of access to clinical trials for targeted therapies. Further research is needed to assess if the broad application of NGS results in better outcomes compared to standard on-site molecular testing while considering the higher cost that may be associated with this approach [60]. Strategies such as enrollment in basket trials and the development of novel targeted drugs are necessary to improve the detection of targetable genomic alterations and to improve access to matched treatment.

## Figures and Tables

**Table 1:** Patient demographic data.

Succesfully Biopsied Patients, *n* = 464 (%)	
Age of tissue (weeks)	
Median	32
Range	0–876
Tissue of origin	
Primary	271 (58.4%)
Metastatic	190 (40.9%)
Unknown	3 (0.6%)
Pretreated	
Yes	155 (33.4%)
No	293 (63.1%)
Unknown	16 (3.4%)
Age	
Median	63
Range	19–88
Gender	
Female	226 (48.7%)
Male	238 (51.3%)
Metastasized	
Yes	335 (72.2%)
No	117 (25.2%)
Unknown	12 (2.6%)
Tumor Group	
Breast	49 (10.6%)
Central nervous system	40 (8.6%)
Carcinoma of unknown primary	16 (3.4%)
Gastro-intestinal	155 (33.4%)
Genito-urinary	72 (15.5%)
Gynaecological	53 (11.4%)
Head and neck	16 (3.4%)
Lung	7 (1.5%)
Melanoma	11 (2.4%)
Mesothelioma	3 (0.6%)
Neuro-endocrine carcinoma	8 (1.7%)
Sarcoma	32 (6.9%)
Thymoma	2 (0.4%)
Tumor Type	
Adrenal gland	1 (0.2%)
Appendix	1 (0.2%)
Bladder	22 (4.7%)
Breast	49 (10.6%)
Cervix	5 (1.1%)
Cholangiocarcinoma	28 (6.0%)
Central nervous system	40 (8.6%)
Colorectal	44 (9.5%)
Carcinoma of unknown primary	17 (3.7%)
Esophageal and gastric	23 (5.0%)
Galbladder	4 (0.9%)
Gastric	10 (2.2%)
Head and neck	8 (1.7%)
Kidney	16 (3.4%)
Liver	3 (0.6%)
Melanoma	11 (2.4%)
Mesothelioma	5 (1.1%)
Neuro-endocrine carcinoma	9 (1.9%)
Non-small cell lung cancer	3 (0.6%)
Ovarian	28 (6.0%)
Pancreas	39 (8.4%)
Parotis	3 (0.6%)
Penis	1 (0.2%)
Prostate	25 (5.4%)
Sarcoma	32 (6.9%)
Small cell lung cancer	2 (0.4%)
Small intestine	1 (0.2%)
Thymoma	2 (0.4%)
Thyroid	5 (1.1%)
Urothelial	7 (1.5%)
Uterine	19 (4.1%)
Vulva	1 (0.2%)

**Table 2:** Classification of alterations according to OncoKB.

Tumor Type	AKT1	ARID1A	BRAF	CDKN2A	EGFR	ERBB2	ESR1	EWSR1	FGFR2	FGFR3	HRAS	IDH1	KRAS	NRAS	PIK3CA	PTEN
Breast	3A	4				1	3A		4						1, 2	
Central nervous system			4		4							3A				4
Carcinoma of unknown primary				3A									4			
Cholangiocarcinoma			2						1			1	4			
Colorectal			1			2							3A, 4			
Esophageal and gastric		4				1			4							
Galbladder													4			
Pancreas													3A, 4			
Small intestine													4			
Bladder										1			4			
Kidney			4													
Penis													4			
Prostate	3A															
Urothelial											3A		4			
Cervix													4			
Ovarian	3A	4											4			4
Uterine									4				4			4
Melanoma			1, 3A											3A		
Neuro-endocrine cancer																4
Sarcoma								4								4
Thyroid			1													4

## Data Availability

Data are available upon reasonable request.

## Supplementary Materials

The following supporting information can be downloaded at: https://www.mdpi.com/article/10.3390/diagnostics13091619/s1, Supplementary Table S1 Frequency of gene alterations, tumor mutational burden, and microsatellite instability.

## References

[1] Slatko B.E., Gardner A.F., Ausubel F.M. (2018). Overview of Next-Generation Sequencing Technologies. Curr. Protoc. Mol. Biol..

[2] Mateo J., Steuten L., Aftimos P., André F., Davies M., Garralda E., Geissler J., Husereau D., Martinez-Lopez I., Normanno N. (2022). Delivering precision oncology to patients with cancer. Nat. Med..

[3] Novel Drug Approvals for 2021. https://www.fda.gov/drugs/new-drugs-fda-cders-new-molecular-entities-and-new-therapeutic-biological-products/novel-drug-approvals-2021.

[4] List of EMA-Approved Medicines. https://www.ema.europa.eu/en/medicines/ema_group_types/ema_medicine/field_ema_web_categories%253Aname_field/Human/field_ema_med_status/authorised-36.

[5] Andre F., Mardis E., Salm M., Soria J.C., Siu L.L., Swanton C. (2014). Prioritizing targets for precision cancer medicine. Ann. Oncol..

[6] Doebele R.C., Drilon A., Paz-Ares L., Siena S., Shaw A.T., Farago A.F., Blakely C.M., Seto T., Cho B.C., Tosi D. (2020). Entrectinib in patients with advanced or metastatic NTRK fusion-positive solid tumours: Integrated analysis of three phase 1-2 trials. Lancet Oncol..

[7] Loriot Y. (2022). Tumor agnostic efficacy and safety of erdafitinib in patients (pts) with advanced solid tumors with prespecified fibroblast growth factor receptor alterations (FGFRalt) in RAGNAR: Interim analysis (IA) results. J. Clin. Oncol..

[8] Kato S., Kim K.H., Lim H.J., Boichard A., Nikanjam M., Weihe E., Kuo D.J., Eskander R.N., Goodman A., Galanina N. (2020). Real-world data from a molecular tumor board demonstrates improved outcomes with a precision N-of-One strategy. Nat. Commun..

[9] Merlin J.-L., Gilson P., Husson M., Harle A. (2020). Targeted PCR vs. NGS for molecular diagnostic in solid tumors and liquid biopsies. How to choose in real-life. J. Clin. Oncol..

[10] Jusakul A., Cutcutache I., Yong C.H., Lim J.Q., Huang M.N., Padmanabhan N., Nellore V., Kongpetch S., Ng A.W.T., Ng L.M. (2017). Whole-Genome and Epigenomic Landscapes of Etiologically Distinct Subtypes of Cholangiocarcinoma. Cancer Discov..

[11] Milbury C.A., Creeden J., Yip W.K., Smith D.L., Pattani V., Maxwell K., Sawchyn B., Gjoerup O., Meng W., Skoletsky J. (2022). Clinical and analytical validation of FoundationOne^®^CDx, a comprehensive genomic profiling assay for solid tumors. PLoS ONE.

[12] Colomer R., Mondejar R., Romero-Laorden N., Alfranca A., Sanchez-Madrid F., Quintela-Fandino M. (2020). When should we order a next generation sequencing test in a patient with cancer?. EClinicalMedicine.

[13] Mosele F., Remon J., Mateo J., Westphalen C.B., Barlesi F., Lolkema M.P., Normanno N., Scarpa A., Robson M., Meric-Bernstam F. (2020). Recommendations for the use of next-generation sequencing (NGS) for patients with metastatic cancers: A report from the ESMO Precision Medicine Working Group. Ann. Oncol..

[14] Le Tourneau C., Kamal M., Tsimberidou A.M., Bedard P., Pierron G., Callens C., Rouleau E., Vincent-Salomon A., Servant N., Alt M. (2016). Treatment Algorithms Based on Tumor Molecular Profiling: The Essence of Precision Medicine Trials. J. Natl. Cancer Inst..

[15] Karol D., McKinnon M., Mukhtar L., Awan A., Lo B., Wheatley-Price P. (2021). The Impact of Foundation Medicine Testing on Cancer Patients: A Single Academic Centre Experience. Front. Oncol..

[16] André F., Bachelot T., Commo F., Campone M., Arnedos M., Dieras V., Lacroix-Triki M., Lacroix L., Cohen P., Gentien D. (2014). Comparative genomic hybridisation array and DNA sequencing to direct treatment of metastatic breast cancer: A multicentre, prospective trial (SAFIR01/UNICANCER). Lancet Oncol..

[17] Meric-Bernstam F., Brusco L., Shaw K., Horombe C., Kopetz S., Davies M.A., Routbort M., Piha-Paul S.A., Janku F., Ueno N. (2015). Feasibility of Large-Scale Genomic Testing to Facilitate Enrollment Onto Genomically Matched Clinical Trials. J. Clin. Oncol..

[18] Sohal D.P., Rini B.I., Khorana A.A., Dreicer R., Abraham J., Procop G.W., Saunthararajah Y., Pennell N.A., Stevenson J.P., Pelley R. (2015). Prospective Clinical Study of Precision Oncology in Solid Tumors. J. Natl. Cancer Inst..

[19] Nagahashi M., Shimada Y., Ichikawa H., Kameyama H., Takabe K., Okuda S., Wakai T. (2019). Next generation sequencing-based gene panel tests for the management of solid tumors. Cancer Sci..

[20] FDA (2017). FDA Grants Marketing Approval to FoundationOne CDx In Vitro Diagnostic 2017. https://www.fda.gov/drugs/resources-information-approved-drugs/fda-grants-marketing-approval-foundationone-cdx-in-vitro-diagnostic.

[21] Karlovich C.A., Williams P.M. (2019). Clinical Applications of Next-Generation Sequencing in Precision Oncology. Cancer J..

[22] Medicine F. Technical Specification. https://info.foundationmedicine.com/hubfs/FMI%20Labels/FoundationOne_CDx_Label_Technical_Info.pdf.

[23] Post T.A. (2017). FDA Authorizes MSK-IMPACT Tumor Profiling Assay. https://ascopost.com/News/58263.

[24] Zehir A., Benayed R., Shah R.H., Syed A., Middha S., Kim H.R., Srinivasan P., Gao J., Chakravarty D., Devlin S.M. (2017). Mutational landscape of metastatic cancer revealed from prospective clinical sequencing of 10,000 patients. Nat. Med..

[25] Center M.S.K.C. MSK-IMPACT: A Targeted Test for Mutations in Both Rare and Common Cancers. https://www.mskcc.org/msk-impact.

[26] Jørgensen J.T. (2021). The current landscape of the FDA approved companion diagnostics. Transl. Oncol..

[27] Wakai T. (2017). Precision Cancer Medicine and Super-computing System. Keio J. Med..

[28] Conroy J.M., Pabla S., Glenn S.T., Burgher B., Nesline M., Papanicolau-Sengos A., Andreas J., Giamo V., Lenzo F.L., Hyland F.C.L. (2018). Analytical Validation of a Next-Generation Sequencing Assay to Monitor Immune Responses in Solid Tumors. J. Mol. Diagn..

[29] Chakravarty D., Gao J., Phillips S.M., Kundra R., Zhang H., Wang J., Rudolph J.E., Yaeger R., Soumerai T., Nissan M.H. (2017). OncoKB: A Precision Oncology Knowledge Base. JCO Precis Oncol.

[30] Takeda M., Takahama T., Sakai K., Shimizu S., Watanabe S., Kawakami H., Tanaka K., Sato C., Hayashi H., Nonagase Y. (2021). Clinical Application of the FoundationOne CDx Assay to Therapeutic Decision-Making for Patients with Advanced Solid Tumors. Oncologist.

[31] Gambardella V., Lombardi P., Carbonell-Asins J.A., Tarazona N., Cejalvo J.M., González-Barrallo I., Martín-Arana J., Tébar-Martínez R., Viala A., Bruixola G. (2021). Molecular profiling of advanced solid tumours. The impact of experimental molecular-matched therapies on cancer patient outcomes in early-phase trials: The MAST study. Br. J. Cancer.

[32] Flaherty K.T., Gray R., Chen A., Li S., Patton D., Hamilton S.R., Williams P.M., Mitchell E.P., Iafrate A.J., Sklar J. (2020). The Molecular Analysis for Therapy Choice (NCI-MATCH) Trial: Lessons for Genomic Trial Design. J. Natl. Cancer Inst..

[33] Trédan O., Wang Q., Pissaloux D., Cassier P., de la Fouchardière A., Fayette J., Desseigne F., Ray-Coquard I., de la Fouchardière C., Frappaz D. (2019). Molecular screening program to select molecular-based recommended therapies for metastatic cancer patients: Analysis from the ProfiLER trial. Ann. Oncol..

[34] Coquerelle S., Darlington M., Michel M., Durand M., Borget I., Baffert S., Marino P., Perrier L., Durand-Zaleski I. (2020). Impact of Next Generation Sequencing on Clinical Practice in Oncology in France: Better Genetic Profiles for Patients Improve Access to Experimental Treatments. Value Health.

[35] Le Tourneau C., Delord J.P., Gonçalves A., Gavoille C., Dubot C., Isambert N., Campone M., Trédan O., Massiani M.A., Mauborgne C. (2015). Molecularly targeted therapy based on tumour molecular profiling versus conventional therapy for advanced cancer (SHIVA): A multicentre, open-label, proof-of-concept, randomised, controlled phase 2 trial. Lancet Oncol..

[36] Mateo J., Chakravarty D., Dienstmann R., Jezdic S., Gonzalez-Perez A., Lopez-Bigas N., Ng C.K.Y., Bedard P.L., Tortora G., Douillard J.Y. (2018). A framework to rank genomic alterations as targets for cancer precision medicine: The ESMO Scale for Clinical Actionability of molecular Targets (ESCAT). Ann. Oncol..

[37] Prasad V., Vandross A. (2015). Characteristics of Exceptional or Super Responders to Cancer Drugs. Mayo Clin. Proc..

[38] von Itzstein M.S., Smith M.L., Railey E., White C.B., Dieterich J.S., Garrett-Mayer L., Bruinooge S.S., Freedman A.N., De Moor J., Gray S.W. (2021). Accessing Targeted Therapies: A Potential Roadblock to Implementing Precision Oncology?. JCO Oncol. Pract..

[39] Garralda E., Dienstmann R., Piris-Giménez A., Braña I., Rodon J., Tabernero J. (2019). New clinical trial designs in the era of precision medicine. Mol. Oncol..

[40] Reitsma M., Fox J., Borre P.V., Cavanaugh M., Chudnovsky Y., Erlich R.L., Gribbin T.E., Anhorn R. (2019). Effect of a Collaboration Between a Health Plan, Oncology Practice, and Comprehensive Genomic Profiling Company from the Payer Perspective. J. Manag. Care Spec. Pharm..

[41] Mangat P.K., Halabi S., Bruinooge S.S., Garrett-Mayer E., Alva A., Janeway K.A., Stella P.J., Voest E., Yost K.J., Perlmutter J. (2018). Rationale and Design of the Targeted Agent and Profiling Utilization Registry (TAPUR) Study. JCO Precis. Oncol..

[42] ClinicalTrials.gov (2020). A Danish Nationwide Clinical Trial on Targeted Cancer Treatment Based on Genomic Profiling (ProTarget). https://clinicaltrials.gov/ct2/show/NCT04341181.

[43] ClinicalTrials.gov A MolEcularly Guided Anti-Cancer Drug Off-Label Trial (MEGALiT). https://clinicaltrials.gov/ct2/show/NCT04185831.

[44] Drilon A.E., Liu H., Wu F., Chen D., Wilson T.R., Simmons B.P., Barlesi F. (2021). Tumor-agnostic precision immuno-oncology and somatic targeting rationale for you (TAPISTRY): A novel platform umbrella trial. J. Clin. Oncol..

[45] Clinicaltrials.gov A Study to Examine the Clinical Value of Comprehensive Genomic Profiling Performed by Belgian NGS Laboratories: A Belgian Precision Study of the BSMO in Collaboration With the Cancer Centre (BALLETT). https://clinicaltrials.gov/ct2/show/NCT05058937.

[46] Clinicaltrials.gov Initiative for Molecular Profiling and Advanced Cancer Therapy (IMPACT II). https://www.clinicaltrials.gov/ct2/show/NCT02152254.

[47] Clinicaltrials.gov PREcision Medicine in Cancer in Odense, Denmark (PRECODE). https://clinicaltrials.gov/ct2/show/NCT05385081.

[48] Blay J.Y., Corset V., Mastier C., Treilleux I., Le Tourneau C., Italiano A., Delord J.P., Attignon V., Wang Q., Baudet C. (2017). PROFILER 02—A multicentric, prospective cohort study aiming to evaluate the added value of a large molecular profiling panel (315 cancer-related gene panel [FoundationOne]) versus a limited molecular profiling panel (74 cancer-related gene panel [CONTROL]) in advanced solid tumours. Ann. Oncol..

[49] Skamene T., Siu L.L., Renouf D.J., Laskin J.J., Bedard P.L., Jones S.J.M., Ferrario C., Whitlock J., Petrie J., Sullivan P. (2018). Canadian profiling and targeted agent utilization trial (CAPTUR/PM.1): A phase II basket precision medicine trial. J. Clin. Oncol..

[50] Massard C., Michiels S., Ferté C., Le Deley M.C., Lacroix L., Hollebecque A., Verlingue L., Ileana E., Rosellini S., Ammari S. (2017). High-Throughput Genomics and Clinical Outcome in Hard-to-Treat Advanced Cancers: Results of the MOSCATO 01 Trial. Cancer Discov..

[51] Miller R.W., Hutchcraft M.L., Weiss H.L., Wu J., Wang C., Liu J., Jayswal R., Buchanan M., Anderson A., Allison D.B. (2022). Molecular Tumor Board-Assisted Care in an Advanced Cancer Population: Results of a Phase II Clinical Trial. JCO Precis. Oncol..

[52] Hoes L.R., van Berge Henegouwen J.M., van der Wijngaart H., Zeverijn L.J., van der Velden D.L., van de Haar J., Roepman P., de Leng W.J., Jansen A.M.L., van Werkhoven E. (2022). Patients with Rare Cancers in the Drug Rediscovery Protocol (DRUP) Benefit from Genomics-Guided Treatment. Clin. Cancer Res..

[53] Prasad V., De Jesús K., Mailankody S. (2017). The high price of anticancer drugs: Origins, implications, barriers, solutions. Nat. Rev. Clin. Oncol..

[54] Remon J., Dienstmann R. (2018). Precision oncology: Separating the wheat from the chaff. ESMO Open.

[55] Swanton C., Soria J.C., Bardelli A., Biankin A., Caldas C., Chandarlapaty S., de Koning L., Dive C., Feunteun J., Leung S.Y. (2016). Consensus on precision medicine for metastatic cancers: A report from the MAP conference. Ann. Oncol..

[56] McKenzie A.J., Dilks H.D., Jones S.F., Burris H. (2019). Should next-generation sequencing tests be performed on all cancer patients?. Expert Rev. Mol. Diagn..

[57] Schwartzberg L., Kim E.S., Liu D., Schrag D. (2017). Precision Oncology: Who, How, What, When, and When Not?. Am. Soc. Clin. Oncol. Educ. Book.

[58] Marabelle A., Mouraud S. (2019). Cancer patients can be categorized by a uniform and mutually exclusive pattern of expression of PD-1 or PD-L1 on their tumor infiltrating T lymphocytes. J. Immunother. Cancer.

[59] Marcus L., Lemery S.J., Keegan P., Pazdur R. (2019). FDA Approval Summary: Pembrolizumab for the Treatment of Microsatellite Instability-High Solid Tumors. Clin. Cancer Res..

[60] Christofyllakis K., Bittenbring J.T., Thurner L., Ahlgrimm M., Stilgenbauer S., Bewarder M., Kaddu-Mulindwa D. (2022). Cost-effectiveness of precision cancer medicine-current challenges in the use of next generation sequencing for comprehensive tumour genomic profiling and the role of clinical utility frameworks (Review). Mol. Clin. Oncol..

