# Supplementary material for: Comprehensive Genomic Profiling and Therapeutic Implications for Patients with Advanced Cancers: The Experience of an Academic Hospital
Source: Diagnostics (Basel). 2023 May 3;13(9):1619. doi: 10.3390/diagnostics13091619 (PMC10177779; doi:10.3390/diagnostics13091619)
Supplement: Supplementary file 1 [file diagnostics-13-01619-s001.zip › diagnostics-2305898-supplementary.pdf]

Supplemental table S1 - Frequency of gene alterations, tumor mutational burden and microsatellite instability

	Median (range)
**Total number of gene alterations per patient**	**4 (0 - 19)**
Breast	4 (1 - 12)
Central nervous system	6 (0 - 11)
Carcinoma of Unknown Primary	4.5 (0 - 19)
Gastro-intestinal	5 (0 - 19)
Genito-urinary	4 (0 - 15)
Gynaecological	4 (0 - 17)
Head and Neck	4.5 (1 - 18)
Lung	5 (2 - 7)
Melanoma	5 (2 - 10)
Mesothelioma	4 (1 - 4)
Neuro-endocrine carcinoma	4 (1 - 16)
Sarcoma	3 (0 - 13)
Thymoma	2 (1 - 3)
**Number of genes altered per patient**	**4 (0 - 18)**
Breast	4 (1 - 12)
Central nervous system	5 (0 - 11)
Carcinoma of Unknown Primary	4 (0 - 17)
Gastro-intestinal	4 (0 - 18)
Genito-urinary	4 (0 - 15)
Gynaecological	4 (0 - 17)
Head and Neck	4.5 (1 - 17)
Lung	5 (2 - 6)
Melanoma	5 (2 - 9)
Mesothelioma	3 (1 - 4)
Neuro-endocrine carcinoma	4 (1 - 16)
Sarcoma	3 (0 - 13)
Thymoma	2 (1 - 3)
**Tumor mutational burden**	**2.52 (0 - 66.8)**
Missing	n = 45 (9.7%)
Breast	3.78 (0 - 16.4)
Central nervous system	2.52 (0 - 7.57)
Carcinoma of Unknown Primary	5.67 (0 - 66.8)
Gastro-intestinal	2.52 (0 - 13.9)
Genito-urinary	3.78 (0 - 35.3)
Gynaecological	3.78 (0 - 42.9)
Head and Neck	2.76 (1.26 - 12.61)

Lung	2.52 (0 - 8.83)
Melanoma	6.3 (2.52 - 34.04)
Mesothelioma	0 (0 - 2.52)
Neuro-endocrine carcinoma	1.89 (0 - 8.83)
Sarcoma	1.26 (0 - 5.04)
Thymoma	2.52 (2.52 - 2.52)
Adrenal gland	1.26 (1.26 - 1.26)
Appendix	NA
Bladder	6.3 (0 - 35.3)
Breast	NA
Cervix	3.78 (2.52 - 8.83)
Cholangio	NA
Central nervous system	NA
Colorectal	NA
Carcinoma of Unknown Primary	6.3 (0 - 66.82)
Esophageal and Gastric	NA
Galbladder	NA
Gastric	NA
Head and Neck	3.78 (1.26 - 12.61)
Kidney	NA
Liver	2.52 (1.26 - 3.78)
Melanoma	6.3 (2.52 - 34.04)
Mesothelioma	0 (0 - 2.52)
Neuro-endocrine carcinoma	2.52 (0 - 8.83)
Non-small cell lung cancer	2.52 (2.52 - 3.78)
Ovarian	NA
Pancreas	NA
Parotis	1.26 (1.26 - 10.09)
Penis	20.17 (20.17 - 20.17)
Prostate	NA
Sarcoma	1.26 (0 - 5.04)
Small cell lung cancer	7.57 (6.3 - 8.83)
Small intestine	8.83 (8.83 - 8.83)
Thymoma	NA
Thyroid	3 (1.26 - 3.78)
Urothelial	NA
Uterine	NA
Vulva	2.52 (2.52 - 2.52)
**Tumor mutational burden high versus low**	**n (%)**
High	42 (9.1%)

Low	377 (81.3%)
Missing	45 (9.7%)
**Microsatellite instability**	**n (%)**
Instable	8 (1.7%)
Stable	404 (87.1%)
Unknown	6 (1.3%)
Missing	46 (9.9%)
